# Environmental Effects on the Ecological Carrying Capacity of Marine Ranching in the Northern South China Sea

**DOI:** 10.3390/biology14040419

**Published:** 2025-04-14

**Authors:** Ziwen Wang, Lijun Yao, Jing Yu, Yuxiang Chen, Xue Feng, Pimao Chen

**Affiliations:** 1 South China Sea Fisheries Research Institute, Chinese Academy of Fishery Sciences, Guangzhou 510300, China; w_ziwen@126.com (Z.W.);; 2 Key Laboratory of Marine Ranching, Ministry of Agriculture and Rural Affair, Guangzhou 510300, China; 3 National Digital Fisheries (Marine Ranching) Innovation Sub-Center, Key Laboratory of Marine Ranching Technology, Chinese Academy of Fishery Sciences, Guangzhou 510300, China; 4 Scientific Observing and Experimental Station of South China Sea Fishery Resources and Environments, Ministry of Agriculture and Rural Affairs, Guangzhou 510300, China; 5 Department of Optoelectronic Engineering, Jinan University, Guangzhou 510632, China; tylj@jnu.edu.cn; 6 Southern Marine Science and Engineering Guangdong Laboratory (Zhuhai), Zhuhai 519000, China

**Keywords:** environmental effects, generalized additive models, marine ranching, South China Sea

## Abstract

The marine ecological carrying capacity (MECC) of marine ranching serves as a crucial indicator for assessing the conservation effect of fishery resources and forms a significant basis for scientific management of coastal fisheries. The environmental impacts on the MECC of marine ranching in the northern South China Sea were analyzed quantitatively by employing Generalized Additive Models (GAMs), which have been successfully applied to the study of the relationship between fishery resources and environmental factors, and factor analysis, using satellite and survey observations. Results showed that 95.40% of the total variation in MECC was explained by these factors. Based on the GAMs, the most important factor was Year (calendar years), with a contribution of 66.20%, followed by Chlorophyll a concentration (Chl-a), Sea Surface Temperature (SST), Dissolved Inorganic Nitrogen (DIN) and Water Current, with contributions of 20.60%, 4.40%, 3.60%, and 0.60%, respectively. The findings of this study inspire us to establish a long-term marine ranching resource and environment monitoring platform, and an early warning and forecasting expert decision-making system, providing scientific references for planning and management of coastal marine ranching.

## Simple Summary

This study used Generalized Additive Models (GAMs) to assess how environmental factors influence Marine Ecological Carrying Capacity (MECC) in the northern South China Sea, finding that 95.4% of MECC variation was explained by key drivers. The most influential factors were Year (66.2%), Chlorophyll-a (20.6%), Sea Surface Temperature (4.4%), Dissolved Inorganic Nitrogen (3.6%), and Water Current (0.6%). The results support long-term monitoring and early warning systems to improve sustainable management of marine ranching fisheries.

## 1. Introduction

Marine Ecological Carrying Capacity (MECC) is an important means of evaluating the conservation status of fishery resources in marine ranching, providing a crucial basis for scientific management of marine ranching [1,2]. Exceeding the capacity of the marine environment poses a serious threat to marine and coastal ecosystems, as well as to the health of coastal residents, causing undesirable effects such as seasonal hypoxia in the marine ranching area, destruction of the original ecological corridor by artificial reef construction, and potential long-term negative impacts of large-scale rapid promotion of marine ranching construction on the original marine ecology and environment [3], even leading to eutrophication, red tides, loss of biodiversity, mass mortality, malformations of marine fish, and the birth of benthos [4,5,6,7,8]. In the construction of marine ranching, MECC may show some fluctuations due to direct or indirect pressures; however, the factors by which they are affected are often uncertain. In Jiaozhou Bay, China, the input of land source pollutants and shoreline destruction caused by human activities are the major reasons for the overloading of the marine carrying capacity in this area [9]. In contrast, socio-economic development indicators such as total imports and exports, port cargo throughput, scientific research and technical services, and the number of employees in the geological exploration industry affect the MECC in the Beibu Gulf, China [10]. Studies have shown that insufficient environmental protection and exploitation of resources in marine ranching ecosystems lead to excessive dependence on external resources, limiting the system’s ability to develop sustainably and thus impacting the ecosystem stability and resilience [11]. Previous studies have focused on the qualitative evaluation of environments on MECC; its quantitative analysis still remains unclear and needs a comprehensive application of various technical means. A quantitative analysis of MECC is needed to identify influencing factors in marine ranching. This analysis should involve a scientific evaluation of MECC and a systematic identification of the factors [12]. Early studies on MECC were closely associated with fishery resource carrying capacity research, using Ecopath models [1], numerical models [13], and improved logistic simulations [14], etc. [15] to calculate the thresholds of marine organisms. In recent years, the evaluation of MECC, influenced by multiple factors, has attracted attention. There are two major directions for index system construction. The first is to build an indicator system based on the overall response of the ecosystem according to the Pressure-State-Response (PSR) model [16]. Models such as the Driving-Pressure-Status-Influence-Response (DPSIR) [10] model and the Driving-Pressure-Status-Influence-Response-Management (DPSIRM) [17] model were developed. The second is to classify the components of the ecosystem, and indicators are selected from various aspects such as society, economy, resources, environment and technology [18]. Most studies that evaluated the factors affecting MECC were based on the established indicator system, which relied on the score of each indicator. Additionally, the obstacle degree model [19] has also been applied to analyze the major influencing factors. Our previous research has shown that marine ranching significantly improves the marine ecological carrying capacity, with resource and environmental factors being the primary drivers. However, the specific contributing variables (or elements) within these factors, as well as their operational mechanisms, remain unclear and require further quantitative analysis.

The relationship between the MECC and its influencing factors is nonlinear and complex, making modeling a powerful tool for research [20]. Generalized Additive Models (GAMs), a nonparametric extension of Generalized Linear Models (GLMs), reveal the nonlinear relationships between response variables and explanatory variables in a highly flexible way with nonparametric smoothing [21], making it particularly suitable for capturing the dynamics between MECC and its influencing factors effectively. GAMs have been widely used to study the relationship between fishery resources and environmental factors, as well as to analyze the spatial and temporal distribution of water quality parameters and the correlation among parameters [22,23,24]. In addition, influencing factors such as ocean flow fields, temperature, biodiversity, water quality, and human activities over time [9], and an accurate assessment of MECC need to be taken into account in these complex spatial and temporal processes. Satellite remote sensing provides large-scale, long-term, and dynamic observations that are an effective complement to survey observations.

This study aims to identify the environmental drivers and their quantitative contributions to the MECC of marine ranching. Therefore, Gaofen satellite and field investigation data from 15 years were used to construct GAMs. The relative importance of environmental and resource variables, including Year, Chlorophyll a concentration (Chl-a), Sea Surface Temperature (SST), Dissolved Inorganic Nitrogen (DIN) and Water Current, were also analyzed. By quantifying nonlinear relationships between environmental factors and ecological carrying capacity, targeted management actions can be implemented to prevent ecosystem collapse. The high-resolution satellite data detect micro-scale variations, which, in turn, support precision management strategies tailored to specific ecological zones. Furthermore, the development of predictive frameworks allows simulation of ecological changes, empowering stakeholders to address risks by adjusting quotas or relocating aquaculture operations. By ranking environmental drivers based on their contribution, the study optimizes resource allocation, ensuring interventions prioritize high-impact factors. This study advances ecological research by transitioning from descriptive assessments to mechanistic and predictive insights through the integration of GAMs and high-resolution satellite data. 

## 2. Materials and Methods

### 2.1. Research Area

**Figure 1:**
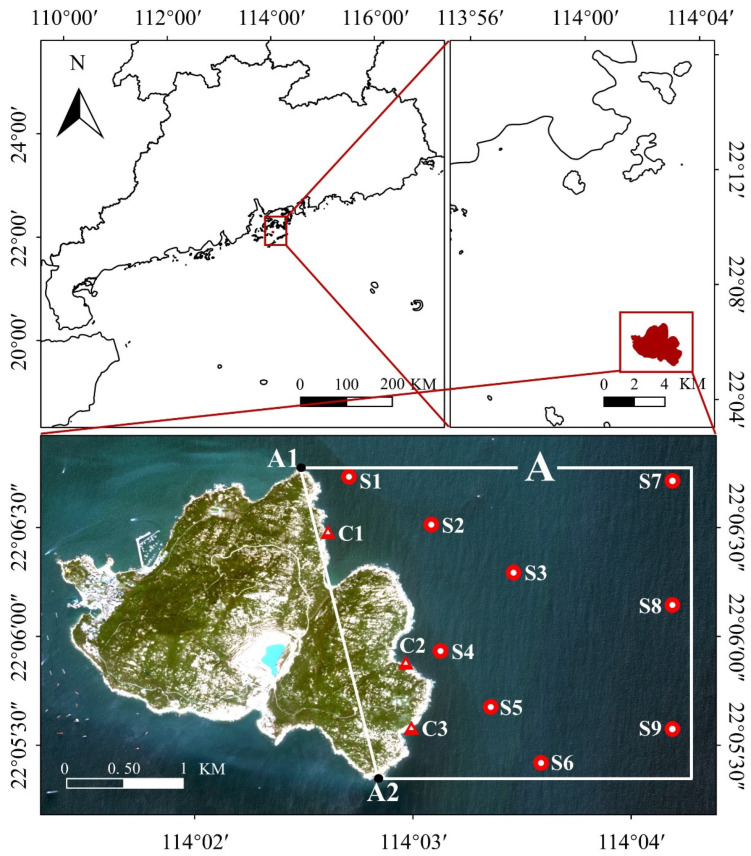
Research area and survey stations.

The research area is in the eastern part of the marine ranching in Wailingding Island and its adjacent intertidal area (Figure 1). The first reef deployment in this area occurred from February 2007 to February 2009, and the second reef deployment took place in 2021; a total of 2937 reefs have been deployed to date. Major organisms conserved are *Sparidae*, *Epinephelus* spp., *Arius sinensis Lacepede*, *Penaeus monodon*, *Sepiella maindroni*, as well as coral and seaweed. A total of 12 survey stations were set up in the research area (Figure 1) to survey water quality, sediment, environmental factors, and fishery resources, along with intertidal benthos in April and September 2020–2021, respectively. Of these stations, S1–S6 and S8 were set to investigate water quality, sediment, environmental factors, and fishery resources. Stations S7 and S9 were set to investigate water quality, and stations C1–C3 were set to investigate intertidal benthos.

### 2.2. Data Processing

Water quality, including temperature, depth, salinity (‰), dissolved inorganic nitrogen (DIN), reactive phosphate (${PO}_{4}^{3}-P$), and marine organisms, including intertidal benthos, phytoplankton, zooplankton, swimming organisms, and fish larvae, were studied. The survey and analysis of samples were carried out in accordance with the Specification for Marine Monitoring (GB17378-2007) [25] and the Specification for Oceanographic Survey-Marine Biological Survey (GB12763.6-2007) [26].

The high-resolution satellite remote sensing data, including Chlorophyll a concentration (Chl-a), Secchi Disk Depth (SDD), Sea Surface Temperature(SST), Water Current, and intertidal data, were derived from the China HY-1 C satellite (https://osdds.nsoas.org.cn/, (accessed on 10 March 2023)). These data, used from January 2019 to October 2022, have a spatial resolution of 50 m for Chl—a and SDD, and 1 km for SST. Water Current data were sourced from the Global Ocean Physical Reanalysis Product of the Copernicus Marine Environment Management Service (CMEMS, http://marine.copernicus.eu/, (accessed on 10 March 2023)), with a spatial resolution of 0.083° × 0.083° and a daily temporal resolution. The data used in this study were extracted for the years 2006, 2009, 2016, 2020, and 2021. Five remote sensing images were selected for intertidal area data (Table 1).

MATLAB 2019 b was used to process satellite remote sensing data, which included resampling to unify data resolution, removing invalid values, and calculating yearly averages. ENVI software 5.6.2 was used to derive the length of coastline, specifically for the land area between points A1 and A2 within the boxed region A in Figure 1. The FVCOM tidal model was used to compute the tidal elevation at the time of satellite overpass. These elevation data were subsequently input into a tidal level calculation formula [27] to extract the intertidal zone. Finally, the coastline length, island area, and beach area were calculated using ArcGIS 10.3 software (Esri, Redlands, CA, USA).

### 2.3. Methods

(1) Factor analysis

According to the previous research [28], 24 variables were selected for this study (Appendix A), namely, natural shoreline (D1, shoreline between A1 and A2 in box A in Figure 1), beach retention rate (D2), island area (D3), vegetation coverage (D4), biomass (D5), biodiversity index (D6), open aquaculture area (D7), artificial reef area (D8), dissolved inorganic nitrogen concentration (D9), reactive phosphate concentration (D10), temperature (D11), salinity (D12), depth (D13), water current velocity (D14), species of fish eggs and larvae (D15), benthic biomass (D16), benthic biodiversity index (D17), zooplankton biomass (D18), zooplankton biodiversity index (D19), phytoplankton density (D20), phytoplankton biodiversity index (D21), nekton density (D22), nekton diversity index (D23), and Chl-a (D24).

Factor analysis was used to simplify multiple variables, revealing the structure behind the data and reducing the complexity of the data while still retaining important information [29]. Initially, Spearman correlation analysis was conducted, followed by a two-tailed *t*-test to assess the significance of the correlation coefficients at the 0.05 and 0.01 levels. The independence between the indicators was then tested by the rank correlation coefficient to achieve a preliminary categorization of the indicators. The calculation of the rank correlation coefficient is detailed in Equation (1) [30].

(1) $$r_{i}=1-6\sum d_{i}^{2}/n\left(n^{2}-1\right)$$

where n is the number of rank pairs of variables x and y, i.e., the sample content, and $d_{i}$ is the difference between ranks of the same pair (*i =* 1, 2, 3, ……, *n*).

Indicators are further categorized through factor analysis by grouping several closely related variables into a single factor that summarizes most of the information in the original set [30] (Equation (2)).

(2) X=AF+ε

where X is the original vector of variables, A is the matrix of the metric loadings, F is the vector of the metrics, and ε is the effect of the metrics on the variance of the data, which is negligible.

Factor scores were estimated for F to analyze the extent to which the indicator contributed to each category of the common factors [30] (Equation (3)).

(3) $$\hat{F}={\left(A^{T}D^{-1}A\right)}^{-1}A^{T}D^{-1}X$$

where D is the special factor variance matrix.

(2) Generalized Additive Models

Based on the MECC obtained from our previous study [28], GAMs were applied to analyze the major influencing factors. The ‘mgcv’ package of software R v.4.0.0 was used to construct and validate the GAMs [31], and the stepwise regression method was applied to select the factors that had a significant effect on the model (Equation (4)).

(4) $$\log\left(MECC\right)\sim \;s\;\left(Year\right)+s\;\left(Chl-a\right)+s\;\left(SST\right)+s\;\left(Current\right)+s\;\left(DIN\right)+\varepsilon$$

where MECC is the marine ecological carrying capacity, s is the function of smoothing splines, s(Year) is the effect of year, s(Chl−a) is the effect of Chl-a, s(SST) is the effect of SST, s(Water Current) is the effect of water current, s(DIN) is the effect of DIN, and ε is the model error that obeys a Gaussian distribution.

(3) Model testing

The Akaike Information Criterion (AIC) was used to test the fit of the model after the gradual addition of factors; the smaller its value, the better the model fit [22,32]. Model predictor variables were assessed using Generalized Cross Validation (GCV); the smaller the value, the better the model generalization [21]. F-test and chi-square test were applied to assess the significance of the factors and the contribution of the factors to the non-parametric effects, respectively [33].

The AIC is calculated as in Equation (5) [34].

(5) $$AIC=2K-2\ln(\hat{L})$$

where k is the number of parameters in the model and $\hat{L}$ is an estimate of the maximum likelihood function value.

## 3. Results

### 3.1. Spatial and Temporal Distribution of Environmental Factors

**Figure 2:**
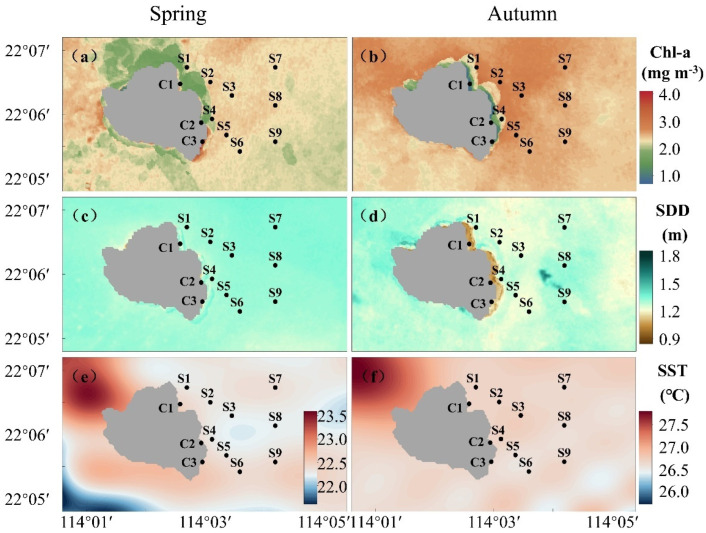
Spatial and temporal distribution of environmental factors in the Wailingding marine ranching: (**a**) Chl−a in spring, (**b**) Chl−a in autumn, (**c**) SDD in spring, (**d**) SDD in autumn, (**e**) SST in spring, (**f**) SST in autumn.

The seasonal distribution of biological environmental factors (Chl-a, SDD, and SST) in the Wailingding marine ranching is shown in Figure 2. Overall, the high Chl-a was in S1–S3 areas. Horizontally, the Chl-a in the marine ranching was higher than that in the surrounding area. The area with high Chl-a expanded and was more evenly distributed in autumn compared to spring. In spring, the Chl-a in the marine ranching ranged from 1.05 to 6.50 $mg\;m^{-3}$, with a mean value of 2.56 $mg\;m^{-3}$. The high values were concentrated at S2, S3, S5, and S8 (Figure 2a), with the maximum recorded at S8 at 2.83 $mg\;m^{-3}$, and the minimum was at S4 at 2.15 $mg\;m^{-3}$. In autumn, the Chl-a ranged from 0.54 to 11.01 $mg\;m^{-3}$, with particularly high values at S2, S3, and S5 (Figure 2b). The peak value was at station S2 with 3.75 $mg\;m^{-3}$ and the lowest value was at S4 with 2.61 $mg\;m^{-3}$. The average concentration for the autumn season was 3.54 $mg\;m^{-3}$, which had an increase of 0.98 $mg\;m^{-3}$ from the spring. In the marine ranching area, Chl-a exceeded 2.50 $mg\;m^{-3}$ at all stations.

Seasonal variations in SDD existed, with an overall trend of low southwest and high northeast in the spring (Figure 2c), and a widening of the area of low SDD and a shift from northeast to southwest in the fall compared to the spring (Figure 2d). The SDD was higher in the spring ranching area, fluctuating between 0.90 and 1.45 m with an average of 1.45 m. The maximum was at S7, which reached 1.39 m, and the minimum was at S4, which was 1.20 m. In autumn, the SDD ranged from 0.92 to 1.60 m, with a mean value of 1.36 m. The values decreased at S2, S3, S5, and S6, with the highest value at S8 at 1.31 m and the lowest value at S6 at 1.15 m.

Seasonal differences in SST were evident (Figure 2e,f). In spring, the SST in the marine ranching ranged from 22.50 °C to 24.00 °C, with an average of 23.13 °C. A cooler region was observed between S3, S4 and S8, while the SST in other regions were distributed evenly. In autumn, the SST was significantly elevated compared to spring, ranging from 26.00 °C to 30.02 °C, with an average of 28.00 °C and a uniform distribution.

### 3.2. GAM Analysis

Due to the repetition and redundancy among the 24 selected indicators, D6, D9, D11, D12, D14, and D24 were selected to be added to the GAMs based on the factor analysis, with a cumulative contribution of 91.71%, which could explain most of the original data.

The cumulative explanatory bias of time and environmental factors on MECC was obtained by using the GAMs fit and predicting the effect of adding variables to the model (Table 2). The selected factors included Year, Chl-a, SST, Water Current, and DIN. The cumulative explanatory deviation of GAMs on the MECC of the marine ranching was 95.40%, with a correlation coefficient $R^{2}$ of 0.95.

In GAMs, the contribution of the selected factors to the MECC of marine ranching indicates a degree of influence (Table 3). The most influential factor was Year, with a contribution of 66.20%, followed by Chl-a, with a contribution of 20.60%. The factors with lesser influence on the model were SST, Water Current, and DIN, with contributions of 4.40%, 3.60%, and 0.60%, respectively. The F-test showed that Year, Chl-a, SST, DIN, and Water Current were significantly correlated with the MECC (*p* < 0.05). The chi-square test showed that the non-parametric smoothing effects of the selected explanatory variables were good.

**Figure 3:**
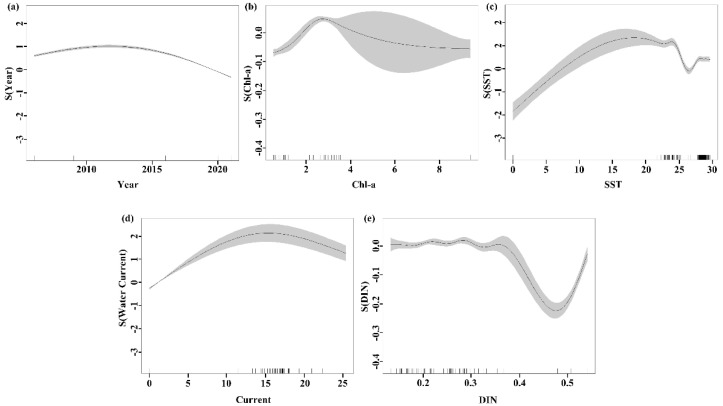
GAMs analysis of factors influencing MECC (**a**) Year, (**b**) Chl−a, (**c**) SST, (**d**) Water Current, (**e**) DIN. Shaded gray areas represent the 95% confidence interval. The x-axis denotes observed values of explanatory variables, while the y−axis shows the smoothed fit of the explanatory variables.

The results of GAMs indicated that Year has the greatest effect on the MECC. During 2009–2020, the MECC increased and then decreased, reaching the maximum in 2013 (Figure 3a). This trend is characterized by a smaller confidence interval and higher confidence level (Figure 3a). When Chl-a was in the range of 0–3 $mg\;m^{-3},$ the MECC increased with Chl-a, exhibiting smaller confidence intervals and higher confidence. When Chl-a exceeded 3 $mg\;m^{-3}$, the MECC decreased with Chl-a, with larger confidence intervals and lower confidence (Figure 3b).

For SST, MECC increased with SST at <24 °C, with smaller confidence intervals and higher confidence. When the SST was at 24–27 °C, the MECC decreased with increasing SST, with smaller confidence intervals and higher confidence. At SST > 27 °C, MECC increased with SST, with decreasing confidence intervals and increasing confidence levels (Figure 3c).

When the Water Current was less than 17 $cm\;s^{-1}$, the MECC increased with increasing flow velocities, with small confidence intervals and higher confidence. When the velocity was greater than 17 $cm\;m^{-1}$, the MECC decreased with increasing flow velocities, with smaller confidence intervals and higher confidence (Figure 3d).

When DIN was at 0.35–0.50 $mg\;L^{-1}$, the MECC decreased with increasing DIN, with decreasing confidence intervals and higher confidence. When DIN was greater than 0.50 $mg\;L^{-1}$, the MECC increased with higher DIN, characterized by smaller confidence intervals and higher confidence (Figure 3e).

## 4. Discussion

### 4.1. Factors Affecting MECC of Marine Ranching

The MECC includes resource supply capacity, environmental constraint capacity, ecological elasticity and human influence, and its changes are influenced by a series of factors [35]. At a high level of organization, the structure and function of ecosystems are related to the performance of ecological carrying capacity, mainly ecosystem stability and ecosystem elasticity [36,37,38]. Ecological carrying capacity is usually quantified as its limit in terms of instantaneous measurements, such as the number of individuals, biomass, and species. It relies on factors that determine growth, including biomass, area, volume, productivity, food, environmental changes, energy, etc. [39]. Among them, resource and environmental conditions play a decisive role in the stability of ecosystems and the diversity and distribution of biological groups, such as fish in the area, thereby affecting the local ecological carrying capacity [40]. Environmental capacity is the constraint of MECC, and the increase in resources and ecological improvement are the material basis and support [41]. Changes in biomass, biodiversity, and the environment characterize, to some extent, changes in the MECC [42]. The increase in biomass and biodiversity can enhance the stability and carrying capacity of the ecosystem, so its ability to resist external changes and recover after damage is enhanced [43]. Environmental improvements provide suitable habitats for organisms and mitigate the effects of pollutants, which, in turn, elevate the MECC of the marine ranching ecosystem.

One study found that in the 11 marine ranches in Yantai, China, natural factors had a greater impact than social factors, and resource constraints were stronger than environmental constraints [44]. Our previous research on the index system has shown that resources and environment in the intertidal zone have a significant impact on the MECC of marine ranches [28]. In Daya Bay, environmental changes in the sea area caused by reclamation have resulted in the loss of some habitats, leading to a decrease in the carrying capacity of the intertidal zone [45]. However, resource finiteness does not mean that the ecological carrying capacity is stable and unchanging; it exists in a dynamic equilibrium. The resources are changing, but at each new moment, the ecological carrying capacity adapts to new conditions when the influencing factors switch to population relationships (competition, predation, etc.), parasites, diseases, food supply, environmental changes, trophic relationships, and the interactions between these factors [39]. In this study, considering the intertidal area that engaged in frequent material and energy exchanges with marine ranching, 24 variables, including intertidal factors, were selected for GAM analysis. It was all about environmental and ecological factors, and the final results showed that Year, Chl-a, SST, Water Current, and DIN had significant effects on the MECC of marine ranching.

### 4.2. Temporal Effects on the MECC of Marine Ranching

GAMs indicated that Year was the largest contribution among the selected factors (Table 3). The trend in MECC was partly related to the construction of the marine ranching. Artificial reefs were deployed twice at the Wailingding marine ranching, in 2009 and 2020, respectively. After the deployment of artificial reefs, upwelling was generated near the reefs, which brought nutrient-rich bottom water to the well-lit euphotic zone through the process of resuspension and diffusion, accelerating nutrient cycling [46]. On the other hand, the reefs produced shadow effect and flow field effect, which not only provided spawning and shelter habitat for organisms, but also provided rich bait resources for fish, shrimp, shellfish and other species, thus improving the biodiversity of the ecosystem and the complexity of the food web. The structural and functional impacts of artificial reefs extended spatially and temporally [47,48], and the long-term effects gradually accumulate in the ecosystem to enhance the ecological carrying capacity of marine ranching. However, given the limited lifespan of artificial reefs, longer reef casting years will affect the physical stability of the reefs and, thus, their ecological effects, resulting in a slight decrease in the MECC over time.

### 4.3. Environmental Effects on the MECC

The MECC of marine ecosystems is closely linked to primary productivity [49], and its relationship with higher trophic levels is often nonlinear and reflects the biomass production potential of the system. Both physical–chemical and biological factors influence marine primary productivity. Research on primary productivity in the Pearl River Estuary (PRE) has shown that temperature, salinity, dissolved oxygen, nutrients, and the intensity of the thermocline are closely related to Chl-a [50]. In the western part of the South China Sea, primary productivity is related to the monsoon and environmental variations along the coastline [51]. Similarly, the primary productivity in the Xisha–Zhongsha waters in the South China Sea is influenced by salinity, light, nutrients, and the oceanic dynamic processes driven by monsoons [52]. Chl-a contributes the most among the environmental factors (Table 3), as it is an indicator of the growth of phytoplankton in the marine ranching, which provides the initial energy to the ecosystem through photosynthesis. Meanwhile, phytoplankton is also the major feeding source for zooplankton and other marine organisms [53], which is at the bottom of the food web. An increase in phytoplankton can provide more ecological niches and resources to support the feeding, habitation, and reproduction of fish, shrimp, and shellfish in the sea area and to increase the resources of marine ranching [54]. Ecosystems are dominated by top-down influences, with top predators having a significant impact on lower trophic levels, but there is always bottom-up control of energy, so that primary producers determine the extent of the ecosystem and phytoplankton productivity influences the stability of the ecosystem. The changes in Chl-a were partly connected with the fact that some marine organisms with high feeding levels had a lag response to Chl-a, due to their growth and developmental habits, resulting in a phenomenon described as ‘high Chl-a with low biomass, low Chl-a with high biomass’ [55]. In addition, Chl-a not only influences the transparency of seawater but also impacts the vision of marine organisms, with good water quality at 3 $mg\;m^{-3}$, which is favorable for biological predation. Research in the southeastern Brazil waters has shown that squid are more likely to find bait to feed on when Chl-a is low, suggesting that lower concentrations may enhance the visibility necessary for predation [56]. When Chl-a increases significantly, the overgrowth of algae consumes excess oxygen and produces toxic substances, leading to threats to the survival of other marine organisms and negatively affecting the MECC of marine ranching. For instance, when Chl-a in the PRE reaches 6 $mg\;m^{-3}$, there is a risk of algal blooms in the surrounding waters [50].

The contribution of SST is second to Chl-a (Table 3). Suitable water temperature affects both the rate of photosynthesis in phytoplankton [50,54] and all stages of the life history of organisms—such as embryos, juveniles, and adults—as well as life activities, including migration, distribution, and spawning in marine ranching [57,58,59]. Different marine species are suitable for different temperatures, depending on their survival and growth. Among the SSTs in this region, 27 °C is critical (Figure 3c). The major conservation species of the Wailingding marine ranching are *Sparidae*, *Epinephelus* spp., *Arius sinensis Lacepede*, *Penaeus monodon*, *Sepiella maindroni*, as well as coral and seaweed, whose suitable temperature is between 22 °C and 30 °C [60,61,62]. Therefore, as the temperature approaches their suitable survival range, life activities are intensified, leading to a gradual increase in the MECC. When the SST falls within the range of 24 °C to 27 °C, the life activities of organisms are vigorous, and competition becomes more pronounced. The escalation of competition can diminish the MECC of the ecosystem [39]. With heightened life activities, the metabolic rate of organisms increases and consumes more energy, while the rise in temperature reduces the solubility of oxygen [63] and also impacts the rate of photosynthesis, especially at higher temperatures [50], which affects the growth and reproduction of marine organisms, resulting in a reduction in the overall MECC. As the temperature continued to rise, the self-regulation of the marine ecosystem allowed the whole to reach a new balance, and the MECC also increased.

Water currents and wind play a key role in marine ecosystems by influencing SST and salinity, which, in turn, affect the life cycles of marine organisms [64] and the composition and structure of fish communities [65]. Large fishing grounds tend to form around large-scale ocean currents, such as *Illex argentinus* in the southwest Atlantic Ocean, the *Ommastrephes bartramii* in the North Pacific Ocean, and the *Todarodes pacificus* in the waters around Japan [22]. The Wailingding marine ranching, situated in a subtropical monsoon climate [66], experienced significant alterations in the topography after the deployment of artificial reefs, which, in turn, intensified the mixed effect [67]. These modifications positively affected the distribution of phytoplankton and the supplementation of bait organisms in the marine ranching. The upwelling generated by artificial reefs transports nutrients from the bottom to the upper and middle waters [68], improves marine primary productivity, attracts marine organisms to congregate [69], and increases biomass and biodiversity. It promotes the food webs’ complexity and ecosystems, strengthens the stability of the ecosystems and their ability to self-regulate, and improves the MECC. Meanwhile, the turbulent flow areas and eddies generated by artificial reefs blocking ocean currents provide spawning and baiting grounds for pelagic fish [70]. The size of the water current is a crucial factor for fish migration and feeding, and marine organisms tend to gather and inhabit areas with relatively low speed and turbulence in order to reduce the energy consumption for swimming [71,72]. For example, the *U. chinensis* has an aggregation of high CPUE in the southern boundary of the PRE, where the water current velocity is 0.20 $m\;s^{-1}$ [23]. Excessive velocities may lead to nutrient deficiencies in the waters, which may have an adverse impact on the productivity and the stability of the ecosystems.

DIN is a key nutrient and a limiting factor for phytoplankton primary productivity. Its distribution and structural variations are influenced by the degree of seawater mixing. Estuarine dynamics of the PRE and the upwelling from artificial reefs are the major drivers of the Wailingding marine ranching ecosystem [73]. The smallest contribution of DIN to MECC was possible due to the influx of riverine input from the PRE that brings large amounts of nutrients to the Wailingding marine ranching, which has an excess of nutrients and a deficit of phosphorus. Consequently, the key factor limiting primary productivity was the phosphate [74].

### 4.4. Limitations and Implications of the Study

In this research, although a series of valuable conclusions have been obtained, there are still some limitations. Firstly, there are issues with data consistency when it comes from different sources, especially remote sensing data, which have different sensor parameters and observational conditions. Although datasets have been processed to meet the required precision, future studies should prioritize cross-validation of satellite-derived data with in situ field surveys to achieve higher precision. Our analysis mainly focused on the isolated effects of individual variables, without fully considering the possible interactions between them. Therefore, it is advisable to consider incorporating interaction effects between variables and time when constructing models in the subsequent study, or to combine methods such as machine learning, neural networks, etc., to explore more complex models and understand the comprehensive impacts. Additionally, micro-scale analysis of ecosystem structure and functional dynamics remains crucial for a comprehensive understanding of the mechanisms underlying ecological carrying capacity.

## 5. Conclusions

In this study, the influencing factors of the MECC of the marine ranching were analyzed quantitatively based on GAMs. GAMs can explain the data well; they advance method applications by integrating with high-resolution remote sensing data. The findings indicated that the year had the greatest influence on the MECC, with a contribution of 66.20%. Among the environmental factors, Chl-a was identified as the most influential, with a contribution of 20.60%. The present study revealed the major influencing factors of MECC and analyzed the responsive mechanisms of the MECC of marine ranching. It has also extended the application of GAMs and high-resolution remote sensing data to fisheries. By interpreting complex ecological interactions into actionable thresholds and forecasts, this framework bridges data-driven science and practical governance. It offers a transformative blueprint for sustainability across diverse systems, ranging from fisheries and biodiversity conservation to public health and climate resilience. The method provides a robust template for marine sustainability and can be directly adapted to other systems by adjusting input variables and thresholds to align with distinct ecological or operational parameters.

## Figures and Tables

**Table 1:** Remote sensing data.

Number	Data Name	Date	Data Source	Spatial Resolution
1	Chl-a	January 2019~October 2022.	HY-1C	50 m
2	SDD	January 2019~October 2022.	HY-1C	50 m
3	SST	January 2019~October 2022.	HY-1C	1 km
4	Water Current	2006 2009 2016 2020 2021	Global Ocean Physical Reanalysis Product of the Copernicus Marine Environment Management Service	0.083° × 0.083°
5	Intertidal area	10 November 2006.	Landsat-5 TM	30 m
6	Intertidal area	23 August 2009.	Landsat-5 TM	30 m
7	Intertidal area	27 March 2016.	BJ-2	0.8 m
8	Intertidal area	22 July 2020.	GF-1	2 m
9	Intertidal area	28 November 2021.	BJ-3	0.5 m

**Table 2:** Deviance analysis for GAMs fitted to MECC.

Model Factors	Residual Deviance	$R^{2}$	AIC	GCV	Accumulation of Deviance Explained (%)
log(MECC)~NULL	44.51	0.00	−367.72	0.0225	0.00
log(MECC)~s(year)	130.92	0.66	−783.25	0.0077	66.20
log(MECC)~s(Year) + s(DIN)	132.26	0.66	−784.50	0.0077	66.80
log(MECC)~s(Year) + s(DIN) + s(SST)	150.49	0.70	−829.00	0.0068	71.20
log(MECC)~s(Year) + s(DIN)+s(SST) + s(Chl-a)	477.83	0.90	−1258.97	0.0023	91.80
log(MECC)~s(Year) + s(DIN) + s(SST)+s(Chl-a) + s(Water Current)	878.663	0.95	−1492.57	0.0012	95.40

**Table 3:** Contribution and significance test in GAMs.

Variables	d.f.	Contribution (%)	Pr (F)	Pr (Chrisq)	VIF
Year	1.999	66.20	2.2 × 10^−16^ ***	2.2 × 10^−16^ ***	3.00
Chl-a	8.995	20.60	2.2 × 10^−16^ ***	2.2 × 10^−16^ ***	3.66
SST	8.867	4.40	6.166 × 10^−10^ ***	1.705 × 10^−10^ ***	3.38
Water Current	2.000	3.60	2.2 × 10^−16^ ***	2.2 × 10^−16^ ***	2.31
DIN	3.110	0.60	0.0930	0.0911	1.41

Signif. Codes: 0‘***’ 0.05‘.’0.1‘’.

## Data Availability

Data are contained within the article.

## Appendix A

**Table A1:** Selected Variables.

**Variables (D)**
Natural shoreline (D1)
Beach retention rate (D2)
Island area (D3)
Vegetation coverage (D4)
Biomass (D5)
Biodiversity index (D6)
Open aquaculture area (D7)
Artificial reef area (D8)
Dissolved inorganic nitrogen concentration (D9)
Reactive phosphate concentration (D10)
Temperature (D11)
Salinity (D12)
Depth (D13)
Current velocity (D14)
Species of fish eggs and larvae (D15)
Benthic biomass (D16)
Benthic biodiversity index (D17)
Zooplankton biomass (D18)
Zooplankton biodiversity index (D19)
Phytoplankton density (D20)
Phytoplankton biodiversity index (D21)
Nekton density (D22)
Nekton diversity index (D23)
Chl-a (D24)

Equation (A1)

(A1) $$r_{i}=1-6\sum d_{i}^{2}/n\left(n^{2}-1\right)$$

Equation (A2)

(A2) X=AF+ε

Equation (A3)

(A3) $$\hat{F}={\left(A^{T}D^{-1}A\right)}^{-1}A^{T}D^{-1}X$$

Equation (A4)

(A4) $$\log\left(MECC\right)\sim \;s\;\left(Year\right)+s\;\left(Chl-a\right)+s\;\left(SST\right)+s\;\left(Current\right)+s\;\left(DIN\right)+\varepsilon$$

Equation (A5)

(A5) $$AIC=2K-2\ln(\hat{L})$$
